# Head and neck squamous cell carcinoma cell lines have an immunomodulatory effect on macrophages independent of hypoxia and toll-like receptor 9

**DOI:** 10.1186/s12885-021-08357-8

**Published:** 2021-09-03

**Authors:** Tamiko Ishizu, Dominik Eichin, Artur Padzik, Sanni Tuominen, Reidar Grénman, Marko Salmi, Tove J. Grönroos, Johanna M. Tuomela

**Affiliations:** 1 Institute of Biomedicine, University of Turku, Turku, Finland; 2 Medicity Research Laboratory, University of Turku, Turku, Finland; 3 Turku Doctoral Programme of Molecular Medicine (TuDMM), University of Turku, Turku, Finland; 4 Turku PET Centre, University of Turku, Turku, Finland; 5 FICAN West Cancer Research Laboratories, University of Turku, Turku, Finland; 6 Turku Bioscience Centre, University of Turku and Åbo Akademi University, Turku, Finland; 7 Department of Otorhinolaryngology, University of Turku, Turku, Finland; 8 Department of Oncology and Radiotherapy, Turku University Hospital, Turku, Finland

**Keywords:** Immune evasion, Anti-cancer immunomodulation, Innate immune response, TLR9, Macrophage polarization, Hypoxia, Immunoediting, Head and neck squamous cell carcinoma

## Abstract

**Background:**

A low tissue oxygen level, < 1% O_2_, is a typical characteristic inside of solid tumors in head and neck cancer (HNSCC) affecting a wide array of cell populations, such as macrophages. However, the mechanisms of how hypoxia influences macrophages are not yet fully elucidated. Our research aimed to study the effect of soluble mediators produced by hypoxic cancer cells on macrophage polarization. Furthermore, we studied the effect of a hypoxic microenvironment on the expression of tumorigenic toll-like receptor 9 (TLR9) and the consecutive macrophage polarization.

**Methods:**

Conditioned media (CM_NOX_ or CM_HOX_) from cell lines UT-SCC-8, UT-SCC-74A, FaDu, MDA-MB-231 and HaCat cultured under normoxic (21% O_2_) and hypoxic (1% O_2_) conditions were used to polarize human monocyte-derived macrophages. Macrophage polarization was measured by flow cytometry and the production of cytokine mRNA using Taqman qPCR. To study the role of TLR9 in macrophage polarization, the lentiviral CRISPR/Cas9 method was used to establish a stable FaDu^TLR9def^ clone.

**Results:**

Our results demonstrate that the soluble mediators produced by the cancer cells under normoxia polarize macrophages towards a hybridized M1/M2a/M2c phenotype. Furthermore, the results suggest that hypoxia has a limited role in altering the array of cancer-produced soluble factors affecting macrophage polarization and cytokine production. Our data also indicates that increased expression of TLR9 due to hypoxia in malignant cells does not markedly influence the polarization of macrophages. TLR9 transcriptional response to hypoxia is dissimilar to a HIF1-α-regulated LDH-A. This may indicate a context-dependent expression of TLR9 under hypoxia.

**Conclusions:**

HNSCC cell lines affect both macrophage activity (polarization) and functionality (cytokines), but with exception to iNOS expression, the effects appear independent of hypoxia and TLR9.

**Supplementary Information:**

The online version contains supplementary material available at 10.1186/s12885-021-08357-8.

## Background

The main purpose of tissue inflammation is to promote tightly self-controlled processes that protect the host and enable adaptation to external danger. While numerous underlying causes can trigger an inflammatory response, the ideal outcome is always similar: as a result, innate immune cells detect the threat, remove it, and gradually reduce the inflammation. One cell type involved in these processes is macrophages (MΦ). MΦ which originate from yolk-sac progenitors or mature from blood monocytes are responsible for tissue homeostasis, inflammatory responses, and reparative functions post-inflammation [1]. Although the immune system is normally tightly controlled by different chemokines and cytokines, it can still be deceived by malignancies to promote their growth instead of fighting them, for example through the actions of MΦ. Previously, MΦ were crudely classified into two main subgroups: (1) classically activated MΦ (M1) and (2) alternatively activated MΦ (M2). Today, however, it is widely accepted that this maturation process, so-called MΦ polarization, is not a permanent end-point stage, but an adaptive and flexible continuum of variable phenotypes [2–4]. So far, several subcategories of alternatively activated MΦ (M2a, M2b, M2c, and M2d) have been identified based on their surface marker expression, secreted cytokines, and biological function [5, 6]. Macrophage polarization has been studied extensively in a multitude of immunological conditions and it is known to vary depending on the T helper (T_h_) cytokine environment [1, 7–9]. Interest in properties and programming of tumor-associated MΦ (TAM) subgroups has also been steadily increasing in cancer research [10–13]. Notably, the alternatively activated M2a and M2c MΦ are of great interest because of their inherent immunomodulatory and pro-regenerative nature, respectively. They have the potential to induce tumor survival by supporting a T_h_2-environment and the concomitant inhibition of antitumorigenic T_h_1-responses, thereby facilitating cancers to evade destruction. Solid tumors in a variety of cancers are known to dampen the cell-mediated immune response by educating TAMs towards an alternatively activated pro-tumorigenic phenotype [14, 15]. Similarly, research in clinical cohorts of non-small cell lung cancer and breast cancer has demonstrated a correlation between the abundance of alternatively activated TAMs and poor patient prognosis [4, 16].

One particular concern is that rapid growth and poor vasculature in solid tumors typically result in deficient tissue oxygenation. The resulting hypoxia (HOX) activates oxygen-sensing pathways, elicits a transcriptional response, and alters gene expression, consequently promoting tumor growth [17]. Previously Raggi et al. have shown that direct exposure of maturating MΦ to HOX in vitro modified their polarization towards the alternative phenotype [18]. However, some controversy in the field remains as a study with prolyl hydroxylase domain 2 (PHD2)-haplodeficient mice showed that irrespective of their oxygenation levels, all tumors had a similar abundance of TAMs with comparable anti-inflammatory phenotypes [19]. Considering that prevailing MΦ polarization is inseparably linked with the signals from their microenvironment, we investigated the effect of soluble mediators produced by normoxic and hypoxic head and neck squamous cell carcinoma cells (HNSCC) on both MΦ polarization and their function in terms of cytokine production [19, 20].

Furthermore, we and others have previously examined the innate immunity receptor toll-like receptor 9 (TLR9) in multiple cancers and assessed the effects of tissue oxygenation on its expression [21–23]. Our previous findings suggest that a moderate expression of TLR9 can be considered a prognostic factor in triple-negative breast cancer [23]. In addition, our observations in different breast cancer subtypes as well as in glioma cells indicate that a decreased oxygen concentration (5% O_2_) in the microenvironment boosts TLR9 expression significantly [23, 24]. To date, research has not investigated the function of TLR9 as a part of the solid tumor-related inflammatory response during hypoxia; hence, little is known of TLR9-mediated inflammation in HNSCC. While all TLRs generally function as innate immune activators, TLR9 acts as a strong inducer of transcription factor nuclear factor κB (NF-κB). The resulting NF-κB activation initiates the production of pro-inflammatory cytokines, chemokines, and type I interferons (IFNs). Once released, these inflammatory signals activate and recruit innate immune system cells, including TAMs. While cytokines such as interleukin-4 (IL-4), interleukin-6 (IL-6), interleukin-10 (IL-10), and granulocyte-macrophage colony-stimulating factor (GM-CSF) are vital in orchestrating acute inflammatory responses in healthy tissue, they have been shown to provide pro-tumorigenic support during cancer-related chronic inflammation in HNSCC [25]. In part, this is due to their effect on different immune cells such as MΦ. Since MΦ are very versatile and their polarization is a highly flexible process, we therefore additionally investigated if a hypoxic microenvironment together with TLR9-induced soluble mediators from HNSCC cells can modify TAM polarization.

## Materials and methods

### Cell culture

**Fig. 1:**
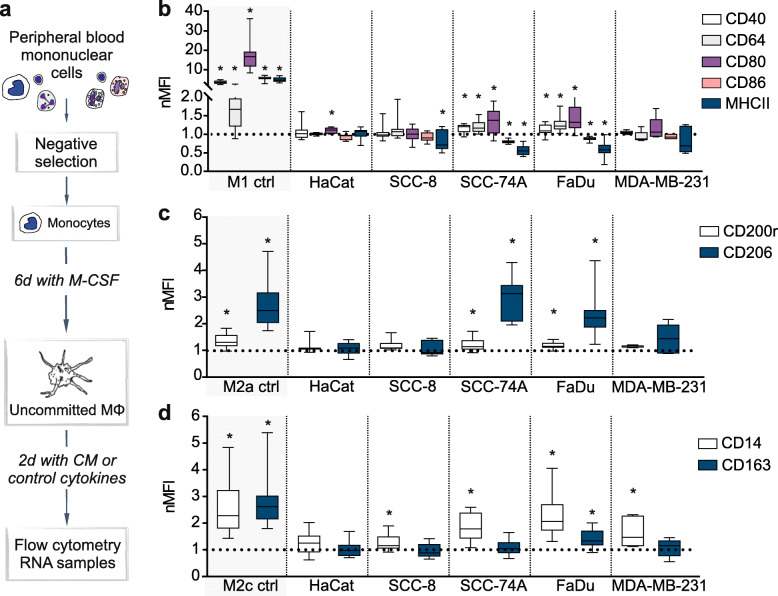
Changes in the relative phenotypical features of non-activated macrophages post conditioned media treatment (**a**) Outline of the conditioned media (CM) -experiment. (**b, c, d**) Following incubation with CM_NOX_ for two days, macrophage (MΦ) polarization was assessed with flow cytometry. Panels **b**, **c**, and **d** illustrate M1, M2a and M2c polarization markers, respectively. UT-SCC-8 and UT-SCC-74A are abbreviated here as SCC-8 and SCC-74A. Box plots represent a comparison of normalized MFI (nMFI) values against NA MΦ (dotted line), error bars 95% CI. Experiments were repeated a minimum of 5 times. *p ≤ 0.05 vs. NA MΦ was considered statistically significant (the exact *p*-values can be found in Additional file F7b)

Two established human UT-SCC-cell lines UT-SCC-8 and UT-SCC-74A (abbreviated as SCC-8 and SCC-74A in Fig. 1) originating from primary, HPV-negative HNSCC tumors, as well as HNSCC cell lines FaDu and FaDu^TLR9def^, keratinocyte cell line HaCat, and breast cancer cell line MDA-MB-231 were investigated in this study. The FaDu^TLR9def^ cells were acquired from Turku Bioscience, Genome Editing Core (establishment of FaDu^TLR9def^ clone is briefly described in Additional Materials and Methods file 1). All cell lines were maintained in Dulbecco’s modified Eagle’s medium (DMEM) with high glucose (Gibco) supplemented with 10% fetal calf sera (Gibco), non-essential amino acids (Gibco), Glutamax (Gibco) and 50 U/mL penicillin and 50 μg/mL streptomycin (Gibco) in a humidified atmosphere at +37°C containing 5% CO_2_. All experiments were performed before primary cell line passages reached 40 doublings to avoid cell transformations due to the culture conditions.

### Preparation of conditioned media and sample collection

Cells were kept in normal atmospheric conditions (NOX, 21% O_2_) or exposed to hypoxia (HOX, 1% O_2_) for up to 2 days and conditioned media (marked onward as CM_NOX_ or CM_HOX,_ respectively) collected from them were used to polarize human primary MΦ in vitro. For the hypoxia treatments, 3 × 10^5^ cells were seeded onto 6 cm^2^ Petri dishes with parallel control cells under normoxia. The next day, the media were replaced with fresh media. Hypoxia incubation took place in the Invivo^2^ hypoxia chamber (Ruskinn Technology Ltd) at + 37 °C. The CMs were collected after 24 and 48 h, centrifuged to remove cell debris, and the supernatants were frozen for the polarization assay. Experiments were repeated 7–10 times, except for MDA-MB-231 where *n =* 5. After the CM was removed from the plate, cells were washed with phosphate-buffered saline before collecting them for immunoblotting or quantitative real-time PCR.

### Human monocyte-derived MΦ polarization

Monocytes from healthy human donors were isolated from mononuclear cells by negative selection using the MACS Monocyte Isolation Kit II (Miltenyi Biotec, (#130–117-337)). Cells were then cultured in Iscove’s Modified Dulbecco’s Medium (IMDM, Gibco) containing macrophage colony-stimulating factor (M-CSF, 25 ng/μl, PeproTech, #300–25) for 6 days to differentiate them towards uncommitted MΦ (MΦ_(M-CSF)_). On day three, half of the medium was changed with fresh IMDM supplemented with M-CSF. On day six, half of the medium (50%) was replaced with CM_(NOX/HOX)_ or CM_(FaDu/FaDuTLR9def)_ for non-activated MΦ_(M-CSF)_ control (marked onwards as NA MΦ) with regular DMEM. The control groups M1, M2a and M2c were induced with LPS (100 ng/mL) + IFNγ (35 ng/mL); IL-4 (10 ng/mL) + IL-13 (35 ng/mL), or TGF-β (15 ng/mL) + IL-10 (15 ng/mL), respectively. After 2 days in culture, the polarization of the MΦ was investigated using flow cytometry and the cytokine production was measured using qPCR (Fig. 1a). Experiments with CM(NOX/HOX) as well as CM(FaDu/FaDu^TLR9def^) were repeated 7–10 times, for MDA-MB-231 *n =* 5.

### Flow cytometry

The expression of MΦ surface markers was assessed with flow cytometry (LSR Fortessa, Becton Dickinson). Unspecific binding on MΦ was blocked with 100 μg/mL human Ig (KIOVIG, Baxter) following staining for 30 min on ice with several primary antibodies: CD14 PE (#555398), CD64 BV510 (#563459), CD68 AF647 (Santa Cruz, #sc-20,060), CD40 BV 510 (#563456), CD80 PE (#557227), CD83 BV421 (#562630), CD86 PerCP Cy5.5 (#561129), HLA FITC (#555558), CD163 PerCP Cy5.5 (#563887), CD200R AF488 (BioRad, MCA2282A488T), CD206 APC (#550889). Unless stated otherwise, antibodies were purchased from Becton, Dickinson, and Company. Fixable Viability Dye eFluor 780 (eBioscience, #65–0865-18) was used as a viability stain. Isotype matched negative control antibodies were used in all stainings (for representative histograms and gating details, see Additional File 2).

### Immunoblot

Following a short sonication, cell lysates in RIPA buffer (supplemented with protease inhibitors; Pierce protease inhibitors, Thermo Scientific) were cleared via centrifugation (10 min, 16,000 x g, + 4 °C). To ensure equal loading of proteins on the electrophoresis gel (SDS-PAGE), the protein concentration was measured by using the bicinchoninic acid method. The Laemmli sample buffer supplemented with dithiothreitol was added and samples were incubated + 95 °C for 5 min to ensure protein denaturation. Samples were run on a gel for ~ 1 h at 100 V. Next, proteins were transferred to a nitrocellulose filter on ice for ~ 2 h at 100 V (Santa Cruz Biotechnologies) before blocking the filter with 5% skimmed milk in tris-buffered saline. Thereafter the membrane was probed overnight with primary antibodies against human TLR9 (Novus Biologicals, #NBP2–24729) or β-actin according to manufacturers’ instructions (Sigma-Aldrich, #A1978) at + 4 °C. After washing with tris-buffered saline/1% Tween, the membrane was incubated with secondary antibody IRDye® 800CW Donkey anti-Mouse IgG (LI-COR Biosciences, # 926–32,212) followed by fluorescent detection with the Odyssey CLx Imager (LI-COR Biosciences).

### Quantitative real-time PCR (qPCR)

Total RNA was isolated according to the manufacturer’s instructions (RNeasy Midi kit, # 75144, Qiagen). RNA concentration was measured with NanoDrop 2000 (Thermo Scientific), followed by the reverse transcription of 1 μg RNA with a mix containing Maxima Reverse Transcriptase (Thermo Scientific, #EP0741), dNTP (Thermo Scientific, #R1121), RiboLock RNase inhibitor (Thermo Scientific, #EO0381) and the oligo-dT mRNA primer (New England BioLabs, #S1316S). 100 ng of cDNA from the in vitro samples was amplified with DyNAmo HS SYBR Green (Thermo Scientific, #F410L) on a CFX96 real-time PCR detection system (Bio-Rad) at a final volume of 20 μl. Similarly, cDNA from polarized MΦ was amplified by quantitative TaqMan Gene Expression Assays using TaqMan Universal Master Mix II, (Thermo Scientific, #4440040) on a 7900HT Fast Sequence Detection System (Applied Biosystems). Finally, the delta-delta Ct (∆∆Ct) method was used for relative quantification of TLR9 and LDH-A gene expression in cancer cells (*n =* 4–6, except for HaCat *n =* 2). Also, cytokine mRNA in CM polarized MΦ was quantified with qPCR. Tata-box binding protein (TBP) was used as a reference gene in both cases. Experiments were repeated 5–7 times, except in the control groups (*n ≥ 2).* The primer information is provided in Additional File 3.

### Quantification and statistical analysis

Data were plotted and analyzed with GraphPad Prism 7.0 software (GraphPad Software). Flow cytometry data were expressed as ratios normalized against NA (mean fluorescence intensity, nMFI). The quantitative PCR data were presented either with ΔCt or normalized ∆∆Ct values. Statistical significance based on nMFI and delta Ct values was calculated with the Wilcoxon Sign Rank test or Kruskal-Wallis test followed by Dunn’s multiple comparison test. The effect of hypoxic CM treatment was analyzed with the Mann-Whitney test. Differences were considered statistically significant when the *p*-value was < 0.05.

## Results

### Conditioned media from HNSCC cell lines induce a mixed M1, M2a and M2c MΦ phenotype

To study MΦ polarization, MΦ maturated from blood monocytes for 6 days were subsequently exposed for 2 days to control cytokines or CM_NOX_ from HNSCC cell lines UT-SCC-8, UT-SCC-74A and FaDu, the breast cancer cell line MDA-MB-231 or a premalignant human keratinocyte cell line HaCat (Fig. 1a). HaCat cells hereby acted as a negative control After two days of exposure, we observed distinct polarization phenotypes in cytokine activated MΦ compared to NA MΦ (Fig. 1b-d). Overall, CMs from UT-SCC-74A and FaDu had the strongest impact on the MΦ phenotype, resulting in an atypical phenotype with mixed M1/M2a/M2c polarization features. The abovementioned cell lines induced the expression of M1 markers CD40, CD64, and CD80, while expressions of CD86 and MHCII were concomitantly decreased in comparison to NA MΦ (Fig. 1b). The upregulation of M2a markers CD200R and CD206 corresponded with positive controls, while only CM from FaDu induced a significant increase of M2c marker CD163 (Fig. 1c,d). Conditioned media from the third HNSCC cell line UT-SCC-8 had a minute impact on the MΦ polarization, as we observed only minor changes in MHCII and CD14 (Fig. 1b,d). To our surprise, in our setting MDA-MB-231 modified moderately the expression of most surface markers, only increasing the expression of M2c marker CD14 (Fig. 1d). In addition, exposure to the CM from the keratinocyte cell line HaCat only mildly increased the expression of the M1 marker CD80 (Fig. 1b).

To confirm a successful polarization, the expression of phenotypic markers was measured in positive control groups generated by LPS + IFNγ treatment for type M1, IL-4 + IL-13 treatment for type M2a and TGF-β + IL-10 treatment for type M2c (as shown in Additional File F4a. Summary of corresponding *p*-values in Additional File F4b). When compared to NA MΦ, the analysis showed a markedly elevated expression of T-cell co-stimulatory molecules CD40, CD80, and CD86 in M1, confirming pro-inflammatory activation. As expected, expression of the same markers remained relatively low or even decreased in the anti-inflammatory M2a and M2c control groups. Expression of MHCII, which enables potentiated antigen presentation in activated MΦ [26] was significantly increased not only in M1 but also in M2a MΦ. In contrast, we detected a significant reduction of MHCII and CD86 expressions in the M2c control. In addition, while the expression of the inhibitory molecule CD200R remained rather limited in the M2a control, its quantity decreased in the M1 control group. Also, CD206, a classical M2a surface marker and non-opsonic receptor related to clearance and presentation of antigens, was significantly increased in the M2a control as expected. Moreover, unlike in the other control groups, only type M2c MΦ had elevated expression of CD14 and CD163, reflecting the distinct type M2c biochemical and physiological profile [27].

### Soluble mediators from normoxic and hypoxic cancer cells promote identical macrophage polarization

**Fig. 2:**
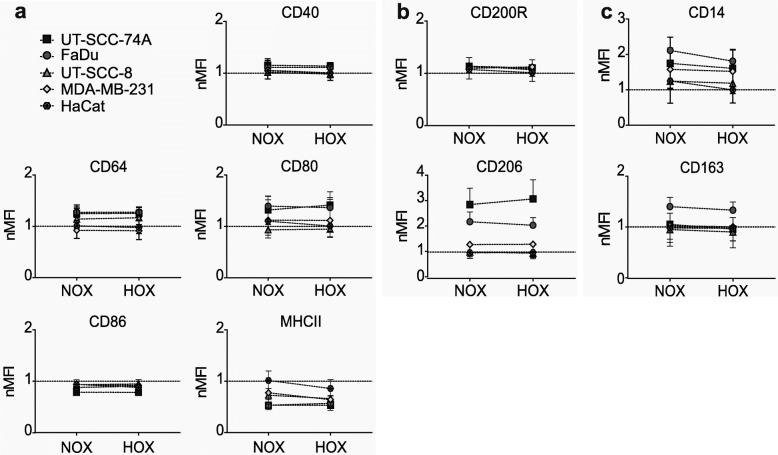
Expression of macrophage surface markers post-incubation with hypoxia-conditioned media (CM_HOX_). Conditioned media collected after 24 h exposure was used to study macrophage polarization. Panel (**a**) shows the resulting change in M1, (**b**) in M2a and (**c**) in M2c marker expression_._ The dotted line represents non-activated macrophages. Results from a minimum of 5 independent experiments are shown as geometric mean of normalized MFI (nMFI) with 95% CI

**Fig. 3:**
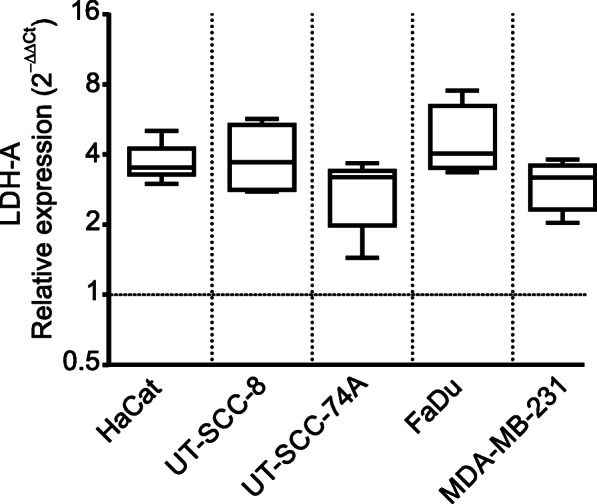
Lactate dehydrogenase A expression under hypoxia compared with normoxia. Succesful hypoxia in cancer cells was verified by measuring hypoxia-inducible factor 1α- regulated lactate dehydrogenase A (LDH-A) expression after 24 h exposure to hypoxia (normoxia, dotted line). Data from a minimum of 4 independent experiments, except HaCat (*n =* 3), is shown as box plots with 95% CI

To evaluate the importance of the microenvironment on cell-secreted soluble mediators, cancer cells were exposed to hypoxic (1% O_2_) conditions before the collection of CM. In comparison to CM_NOX_, CM_HOX_ induced equivalent surface marker expressions across the polarization groups M1 (Fig. 2a), M2a (Fig. 2b), and M2c (Fig. 2c), resulting in negligible changes to MΦ polarization. However, the lack of response was not due to the inability of cancer cells to respond to hypoxia. This was confirmed by measuring the expression of the hypoxia-inducible factor-1 (HIF-1α)- regulated gene lactate dehydrogenase A (LDH-A) expression, which surged at 24 h and persisted at 48 h of hypoxia (Fig. 3). These results suggest that HIF-1α alone has an inconsequential impact on cancer cell-secreted soluble mediators and the resulting MΦ polarization phenotype.

### HNSCC cells reshape the functional phenotype of macrophages independent of hypoxia

**Fig. 4:**
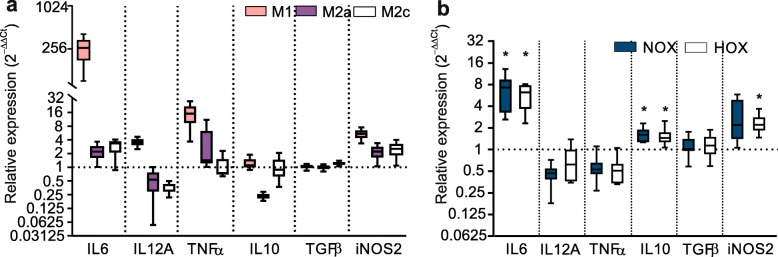
Cytokine mRNA expression in macrophages (MΦ) exposed to conditioned media (CM) Following 2-day exposure to either polarizing cytokines or conditioned media from FaDu cells collected under normoxia (CM_NOX_) or hypoxia (CM_HOX_), MΦ mRNA was extracted and the cytokine expression profile was assessed. Panel (**a**) shows cytokines produced by M1, M2a, and M2c polarization controls. Panel (**b**) shows the cytokine mRNA expression after treatment with CM_NOX_ or CM_HOX_ from parental FaDu cells. Experiments were repeated a minimum of 5 times, except in the control groups (*n ≥ 2).* *p ≤ 0.05 vs. NA MΦ (dotted line), the exact p-values can be found in Additional file 5

Next, we investigated what impact CMs from normoxic and hypoxic HNSCC cells have on MΦ cytokine mRNA production_._ Using qPCR we assessed cytokine mRNA expression in the positive M1, M2a, and M2c controls as well as CM-treated MΦ and compared it to NA MΦ (Fig. 4). The cytokines produced by the polarized control groups were largely expressed as expected (Fig. 4a). M1 expressed markedly all cytokines except IL-10 and TGF-β. While both M2a and M2c groups shared a trend towards increased IL-6 expression and reduced IL-12 levels, only M2a had a decreased expression of IL-10.

Furthermore, we observed clear changes in the cytokine mRNA expression profile in MΦ treated with FaDu CM_NOX_ and CM_HOX_ collected at the 48-h time point (Fig. 4b, the exact *p*-values can be found in Additional File 5a Interestingly, irrespective of the microenvironment, both CMs induced a statistically significant increase in IL-6 as well as IL-10, representing a functional phenotype in MΦ that is between M1 and M2 cells. Albeit none of the remaining cytokines reached a statistical significance when compared to NA macrophages, their expressions were relatively similar to M2 polarized control, showing downregulation of IL-12A, upregulation of inducible nitric oxide synthase (iNOS 2) and no response in TGF-β expression. The only exception was TNF-α, which reduced expression diverged from all controls. Moreover, there was no statistical difference between CM_NOX_ and CM_HOX_ induced MΦ polarization phenotypes.

### TLR9 has a negligible influence on macrophage polarization

**Fig. 5:**
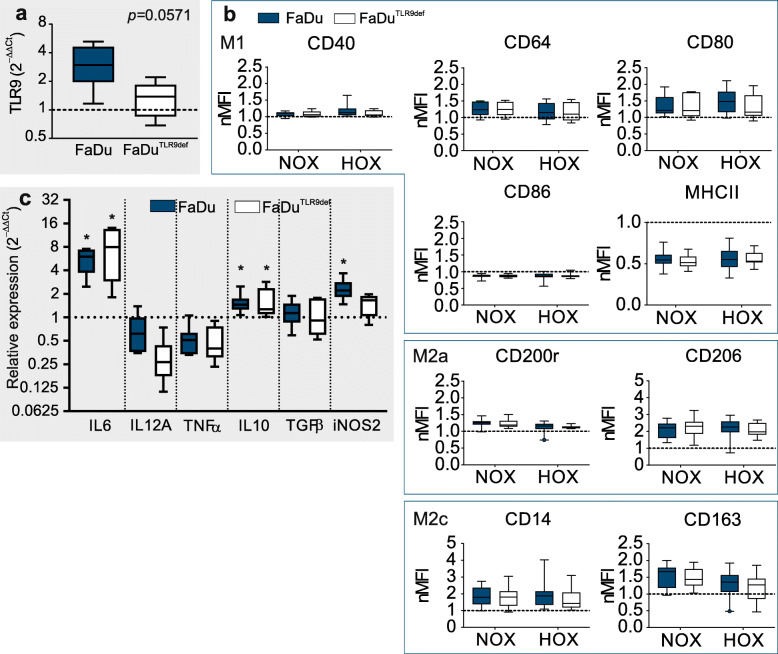
The influence of FaDu and FaDu^TLR9def^ conditioned media on macrophage polarization and mRNA cytokine expression. (**a**) After 48 h of exposure to hypoxia, mRNA from parental FaDu and FaDu^TLR9def^ cells was extracted to assess their toll-like receptor 9 (TLR9) expression (normoxia, dotted line). *p* = 0.0571, *n* = 4. (**b**) Non-activated macrophages (NA MΦ) (dotted line) were treated with conditioned media (CM) for 2 days prior assessing their polarization phenotype with flow cytometry. Panel (**c**) illustrates cytokine mRNA expression from MΦ after treatment with CM from hypoxic parental FaDu or FaDu^TLR9def^ cells. Results are shown as box plots with 95% CI. The dotted line represents NA MΦ. Experiments were repeated a minimum of 5 times. NA vs. CM_HOX_-treated MΦ was considered statistically significant when *p ≤ 0.05. The exact p-values can be found in Additional File 5b

By utilizing TLR9 siRNAs, our previous research has shown that silencing TLR9 in MDA-MB-231 cells enhanced cancer cell viability and their invasive capability under hypoxia [23]. Here we investigated if suppressed TLR9 can influence cancer cell-secreted soluble mediators repertoire and consequently the MΦ polarization under hypoxic conditions. FaDu cells were selected as a model cell line since these cells had shown the highest TLR9 induction under HOX exposure (more details in Additional File F6). mRNA analysis of TLR9-depleted FaDu clone (FaDu^TLR9def^) showed a decreased TLR9 expression under hypoxia in comparison to parental FaDu cells (Fig. 5a, expression under normoxia is shown in Additional Materials and Methods file 1). However, NA MΦ exposed to CM_NOX_ from FaDu^TLR9def^ resulted in polarization equivalent to NA MΦ exposed to CM_NOX_ from parental FaDu cells (Fig. 5b). Moreover, we observed that CM_HOX_ from FaDu^TLR9def^ had a similar impact on surface marker expressions (upregulation of markers CD40, CD64, CD80, CD200r, CD206, CD14, and CD163, as well as downregulation of markers CD86 and MHC II) as CM_HOX_ from parental FaDu cells (Fig. 5b). Thus, in comparison to the hypoxic microenvironment alone, HOX together with reduced TLR9 caused no further alterations in MΦ polarization. Analysis of the functional phenotype resulted in a similar outcome with all CMs. Cytokine mRNA expressions remained largely microenvironment and TLR9-independent when compared to the NA MΦ (Fig. 5c). The only exception was iNOS2 which had a significant increase in expression with CM_HOX_ from parental FaDu, but not from FaDu^TLR9def^ cells in comparison to normoxia. Lastly, IL-12A expression in MΦ exposed to CM_HOX_ from FaDu^TLR9def^ was further downregulated compared to CM_HOX_ from parental FaDu. While this result did not reach statistical significance, it was yet an interesting finding as the expression of IL-12 cytokine is NF-κB-regulated. Together these results suggest that hypoxia-induced TLR9 expression alone is likely not a sufficient immunomodulatory factor regarding TAM polarization and activation.

## Discussion

During an immunological challenge re-shaping of MΦ polarization profoundly affects subsequent MΦ actions in terms of instructing and enhancing the adaptive immunity, resolving inflammation, and repairing tissue damage [28]. In this study, we show that HNSCC-originating cell lines can mount a polarized response and affect the functional phenotype of MΦ by influencing their surface marker expression and cytokine production. As a result, cancer cell-secreted soluble mediators have a significant part in tumor immunoediting; an activity that is known to contribute to tumor progression [14, 20, 29, 30]. Regardless of conventional cancer-related M1-M2 dichotomy described in the literature, we observed an HNSCC cell-induced shift in MΦ polarization towards mixed features of M1/M2a/M2c phenotypes. However, these distinct “hybridized” MΦ phenotypes are not unusual in biological organisms. Several reports describe a variable mix of M2-like homeostatic phenotypes in MΦ, for example in the context of prenatal development, obesity, and infection from parasites or viruses [31–33]. Single-cell transcriptome studies have been of great value in showing that cancer-related MΦ polarization results in immunophenotypes containing a heterogeneous assortment of features present in more than one phenotype [34]. In addition, by analyzing the whole transcriptomes of mouse peritoneal and bone marrow-derived MΦ, Orecchioni et al. showed a more mutual gene expression in apparently opposing polarization phenotypes that have been previously measured [35]. Furthermore, a recent study in a human glioma mouse model described that microglia can obtain heterogeneous phenotypes that do not rigorously follow the classical M1-M2 dichotomy [36]. Besides experimental model-related global changes in MΦ gene expression, also tissue- and cancer subtype-specific signatures have been reported, adding another layer of fluidity to the MΦ transcriptome, especially in vivo [37, 38]. As it happens, these controversial studies challenge researchers to assess how delineative MΦ polarization results obtained in vitro can be to tumors in vivo*.* Therefore, careful interpretation of the polarization results is needed*.*

In this study, we analyzed the outcome of selected cytokines on MΦ polarization only in the control groups. Being limited to this, our study lacks the insight of particular species of molecular mediators in CMs which induce observed MΦ polarization phenotypes. However, several studies have before dissected many of the key mediators HNSCC use to promote pro-tumorigenic MΦ polarization. Regulatory cytokines relevant to MΦ polarization such as IL-1α, IL-4, IL-6, IL-8, IL-10, IL-23, M-CSF, GM-CSF, EGF, PGE_2_ and TGF-β are typically expressed in HNSCC [25, 39, 40]. Moreover, Kesselring et al. have demonstrated in humans that HNSCC tumors induce Th17-promoting milieu which, depending on the context, has been linked with anti-inflammatory features [41]. TAMs have also been shown to resist responding and producing pro-inflammatory cytokines due to their impaired NF-κB signaling [42]. In concert with defective p50, IL-4 together with IL-13 or IL-10, signal via STAT6 or STAT3, respectively, making STATs master regulators of M2 phenotype [43]. Furthermore, several lines of investigation have linked peroxisome proliferator-activated receptor-gamma (PPAR-Ɣ) to be an additional key factor boosting the expression of M2 genes [44–46].

Thus, it is plausible that unregulated and unbalanced expression of some, or many of these, are responsible for developing a mixed M1/M2a/M2c MΦ polarization phenotype we observed. Adding to that, more recent attention has focused on MΦ polarization-altering novel immunoregulatory species such as regulatory miRNA containing exosomes originating from cancer cells or MΦ itself [47, 48].

We discovered that in our experimental setting, CMs from UT-SCC-74A and FaDu were the only cell lines modulating the expressions of the panel of surface markers across polarization phenotypes. Soluble mediators from UT-SCC-8 were not able to modify any other markers than MHCII and CD14. To our surprise, CM from MDA-MB-231 markedly affected only CD14 expression. While Gionfriddo et al. have obtained similar results suggesting largely unaffected MΦ polarization by the breast cancer cell line MDA-MB-231, also contradictory reports have been published [14, 44, 49]. One possible mechanism to the discrepancy can be the different protocols used to isolate, and especially maturate, CD14+ monocytes to MΦ. Yet these results demonstrate that MΦ polarization is more than a bipolar process; it reflects a highly dynamic chameleon-like MΦ phenotype re-programming, responding to the tumor microenvironment-derived stimuli, influenced by the cell-of-origin in cancer (represented in Additional File F7a).

What is more, FaDu was the only cell line to induce a significant change in the M2c marker CD163. The emphasized M2c characteristic is an interesting finding as M2c MΦ have been linked especially with early steps in wound healing, matrix-remodeling, endocytic scavenging, and reciprocal regulation of regulatory T-cells and T_H_2 cells [5, 50, 51]. Moreover, several reports in HNSCC patient cohorts have suggested that CD163+ MΦ could be used as a biomarker in patient prognosis [52]. However, the use of CD163 as a rigid and predictive marker representing exclusively M2- polarization has been challenged by Barros et al. as they showed a global increase of tissue CD163+ MΦ irrespective of predominant Th1- and Th2- immune challenges in situ [53]. They emphasized that CD163+ positivity together with other markers, rather than alone, could be used to characterize MΦ in situ. Thus, this leaves the relevance of CD163 as an M2-marker to be evaluated by care case by case.

Furthermore, a significantly decreased expression of the M1-marker MHCII suggests that HNSCC CM- treated MΦ may have a limited antigen presentation capacity, resulting in insufficient priming and proliferation of CD4+ T helper- cells. Not only have previous studies in mice shown that MHCII^low^ MΦ are more M2-oriented but also discovered that tumorigenic MHCII^low^ MΦ promote angiogenesis and tumor progression [54, 55]. On the other hand, data collected from septic patients by Döcke et al. suggested that loss of MHCII in monocytic cells could be a result of hyperactive counter-regulatory mechanisms. This results in a profoundly immunodeficient phenotype characterized by a reduced capacity to produce pro-inflammatory cytokines, such as TNF-α [56]. Also, our data showed that two out of three CMs induced downregulation of the T- cell co-receptor CD86, demonstrating the overall sedentary effect cancer cells have on MΦ. Importantly, a similar phenotype of CD86^low^/MHCII^low^ MΦ was observed in M2c control induced with TGF-β + IL-10. This agrees with previously reported results as IL-10 in association with TGF-β has been shown to downregulate MHCII and T-cell costimulatory molecules [56, 57].

A substantial body of evidence has established that activation of the HIF-1α pathway is responsible for shaping the phenotype, metabolism, and activity of cancer cells. Previous results in mouse mammary and 3LL lung carcinoma tumor models indicate that hypoxia-driven HIF-1α activation in MΦ advances their polarization towards the anti-inflammatory M2 phenotype [18, 54, 58]. However, our approach utilized CM from hypoxic cancer cells, hence MΦ were not exposed to HOX at any given time. Yet it is somewhat surprising that 1% HOX, in comparison to NOX, did not change the measured polarization parameters in MΦ. Consequently, this raises an interesting consideration of how cancer cells prioritize available survival strategies in a hypoxic microenvironment, like in tumor stroma; our data suggest that enhanced immunoescape is less vital for malignant cells to coordinate than e.g. conducting a swift metabolic switch by increasing glycolysis and cutting back mitochondrial respiration [59]. Another explanation to the unpredicted similarity between CM_NOX_ and CM_HOX_-treated MΦ polarization can be secondary molecular factors, such as lactate, growth factors, insulin, and elevated Th_1_ cytokines; factors which are known to stabilize and increase the translation of HIF-1α independent of physiological HOX [60–63]. In addition, besides HIF-1α, our study did not consider other HIF isoforms with distinct functions and tissue expression, such as HIF-2α, in hypoxia response. Further investigation is needed to determine whether these factors influence cancer cell-induced MΦ polarization.

Despite this, the phenotypes of CM-exposed MΦ correspond to TAMs seen in hypoxic areas inside multiple tumors with different origins. Yet, it is worth considering that although the mechanisms responsible for polarizing MΦ in our in vitro setting suggests it being hypoxia-independent, it does not exclude a possibility that a direct HIF-1α response in MΦ in vivo could be relevant in sensitizing them to respond to external stimuli under stressful conditions. As it happens, a report by Müller et al. utilized single-cell RNA-sequencing to demonstrate that within human gliomas the tissue-resident microglia and blood-derived TAMs are not only obtaining distinct phenotypes but that these cell populations also localize in separate areas. While microglia were found enriched at the leading edge of the tumor, TAMs traveled to the intratumoral hypoxic areas [64]. Thus, tumor hypoxia is likely to serve as a switch for blood-derived myeloid cells to orchestrate pro-tumorigenic functions such as angiogenesis and immune suppression.

Our qPCR data describing the MΦ cytokine profile demonstrates that MΦ exposed to soluble mediators from FaDu cells are functionally more analogous to M2a/M2c controls than M1; a result which once more appears microenvironment-independent. After exposure to FaDu CM MΦ displayed elevated expression of IL-6 mRNA, confirming a functional immunological reaction. Although IL-6 is in general considered a pro-inflammatory cytokine, it can under certain circumstances act as an anti-inflammatory, and consequently pro-tumorigenic factor [65]. Independent studies have confirmed a clinicopathological relevance for this multifunctional cytokine in HNSCC etiology as its abnormal concentration has been linked with poor patient survival [66, 67]. Like IL-6, also IL-10 mRNA expression increased following exposure to CM. This prevalent anti-inflammatory cytokine, a key activator of T-cell mediated activities, has also been reported to be increased in TAMs in a clinical cohort of oral cavity squamous cell carcinoma patients [68]. Interestingly, we detected significantly reduced mRNA levels of IL-10 in our M2a MΦ control. While that was a slightly surprising finding, it might represent a strong negative feedback loop response to an independent cancer cell-secreted factor. Altogether, the relevance of this finding remains to be evaluated in future studies. Furthermore, our data from M2-polarized controls showed reduced amounts of IL-12 and TNF-α mRNAs; a trend that was positively correlated in CM-exposed MΦ. Thus, our observations in terms of cytokine response consolidate the two-way immunomodulatory phenotype MΦ obtain and maintain jointly with HNSCC cancer cells. Despite the promising findings, care should be taken in extrapolating the results to protein levels as the current study only evaluated the expression of cytokines at the gene level.

Our results also suggest that TLR9 has only a minor immunomodulatory function in vitro. The discovery that hypoxic FaDu CM induced a significant iNOS2 expression in MΦ while FaDu^TLR9def^ CM failed to do so is intriguing and indicates that tumor-based TLR9 can influence MΦ function in a specialized way. In accordance with the present results, Pudla et al. have previously demonstrated that TLR9 has a role as a regulator of iNOS2 expression in mouse MΦ [69]. Except for inducing iNOS2, FaDu^TLR9def^ cells did not noticeably alter MΦ polarization compared to parental FaDu cells, consistent with research done by Mella et al [70]. While MΦ were not included in Mella’s study, their research suggested that tumorigenic TLR9 and tumor-infiltrating CD8+ T-cells do not correlate with triple-negative breast cancer (TNBC) patient cohort. This data, therefore, implies that tumor-originating TLR9 may have a more finite immunomodulatory impact than previously thought. Although TLR9 expression in FaDu^TLR9def^ cells was reduced in comparison to parental FaDu under NOX, it seems to have more complex regulation under cellular stress. It is not uncommon that microenvironmental stress alters cellular processes such as transcription, post-transcriptional regulation, and degradation, affecting not only mRNA but also protein half-life. Thus, due to the partial deletion of TLR9 in FaDu cells, these observations remain somewhat limited. Therefore, repeating experiments with homozygous FaDu^TLR9def^ clones may provide a clearer perspective. Moreover, the maximal expression of TLR9 in HNSCC appears to depend on multiple signals delivered to a cell in a defined sequence, resulting in more latent than acute expression under hypoxia. This finding suggests a context-dependent, and temporal regulation of TLR9 and underlines a possibility that its role becomes substantial only under chronic hypoxia. Since TLR9 in HNSCC cell lines does not demonstrate a significant immunoediting function on MΦ, it is possible a similar result would repeat in TNBC. Yet, a note of caution is due here as the seemingly absent association might correlate with cancer-specific characteristics. While our observations in HNSCC may support this hypothesis, results must be extrapolated with caution and supported by more studies.

## Conclusions

In summary, our findings suggest that the immunomodulation of MΦ phenotype and function executed by HNSCC is not directly reinforced by hypoxia-induced soluble messengers. Further, elevated levels of tumoral TLR9 produce dispensable effects on MΦ polarization under HOX.

## Supplementary Information

**Additional file 1. **Additional Material and Methods. TLR9-targeted CRISPR/Cas9 modification in FaDu cells. (a) mRNA analysis verified that in CRISPR/Cas9-modified FaDu cells (FaDu^TLR9def^) TLR9 mRNA expression under normoxia was significantly reduced with median of 0.5105 with 95% CI (± 0.1792 and 1.125) in comparison to parental FaDu cells (dotted line). Error bars represent min and max, *n* = 7, *p ≥ 0.05. (b) Western blot gels showing a moderate decrease of TLR9-protein expression in CRISPR/Cas9-modified FaDu cells. (c) The clone with the lowest TLR9-expression was selected for Sanger sequencing. The sequencing result confirmed that the cell line had a genetic modification in one allele only.

**Additional file 2.** The gating strategy for macrophage analysis. (a) The gating strategy of human monocytes based on their FSC/SSC profiles, following the viability marker Fixable Viability Dye eFluor 780 and anti-CD68+-based subgating. (b) Representative flow cytometry histograms show all treatment groups and controls for a particular antibody: isotype (dark green), NA (red), M1 (blue), M2a (orange), and M2c (light green).﻿ 

**Additional file 3. **Primers used in real-time PCR. 

**Additional file 4.** Non-activated MΦ controls M1, M2a, and M2c polarization marker expressions. (a) Following incubation with control cytokines (M1: LPS + INFγ; M2a: IL4 + IL13; M2c: TGFβ+IL10) for two days, MΦ polarization was assessed with flow cytometry. Box plots represent MFI values normalized to non-activated MΦ (nMFI), error bars 95% CI. The dotted line represents non-activated MΦ. n = 5–10 independent experiments. All markers, except CD80 in M2c, were statistically significant (*p* ≤ 0.05). (b) Listed p-values describing the statistical significance of M1 (green), M2a (blue), and M2c (gray) marker expressions. The tint of the color signifies the direction of the expression: darker color represents a median expression (nMFI) above NA MΦ. Similarly, lighter color represents median expression below NA MΦ. 

**Additional file 5. **Cytokine mRNA median ∆Ct and statistical p-values of MΦ treated with FaDu conditioned media collected under normoxia or hypoxia (CM_NOX_ or CM_HOX_). MΦ were treated with (a) conditioned media from FaDu cells collected under normoxia or hypoxia (CM_NOX_ or CM_HOX_) or (b) conditioned media from FaDu or FaDu^TLR9def^ after 48 h exposure to HOX.

**Additional file 6.** Expression of TLR9 mRNA under hypoxia (a) Cancer cells were exposed to hypoxia (HOX, 1% O_2_), and RNA samples were collected at 24 and 48 h to assess TLR9 mRNA expression. Unlike lactate dehydrogenase-A (LDH-A) mRNA expression, TLR9 mRNA had a variable expression pattern among cell lines. The dotted line represents corresponding normoxic (21% O_2_) samples. Results from a minimum of 3 independent experiments are shown as box plots with 95% CI. (b) Expression of TLR9 may require input from several cooperative factors together with HIF-1α. TLR9 mRNA expression in FaDu cells increased gradually during prolonged HOX exposure.

**Additional file 7. **A summary of the CM-induced MΦ polarization phenotypes (a) CM-mediated hybridized MΦ polarization phenotype, demonstrates a direct mechanism cancer undertakes to evade anti-tumorigenic immune responses. Values represent MFI values normalized to non-activated MΦ (nMFI), *n* = 5–10. (b) Listed p-values describing the statistical significance of M1 (green), M2a (blue), and M2c (gray) marker expressions after exposure to the variety of CMs. The tint of the color signifies the direction of the expression: darker color represents a median expression (nMFI) above NA MΦ. Similarly, lighter color represents median expression below NA MΦ.

## Data Availability

The datasets supporting the conclusions of this article are included within the article and its additional files.

## Abbreviations

MΦ: macrophage
TLR9: Toll-like receptor 9
HNSCC: Head and neck squamous cell carcinoma
CM: Conditioned media
mRNA: messenger RNA
qPCR: quantitative PCR
M1/M2a/M2c: Type 1/2a/2c polarized macrophages
TAM: Tumor-associated MΦ
HIF-1α: Hypoxia-inducible factor 1α
LDH-A: Lactate dehydrogenase A
iNOS2: Inducible nitric oxide synthase 2
TNBC: Triple-negative breast cancer
NF-κB: Nuclear factor kappa B
IL-: Interleukin
GM-SCF: Granulocyte-MΦ colony-stimulating factor
M-CSF: Macrophage colony-stimulating factor
T_h_: T-helper
HOX: Hypoxia
NOX: Normoxia
NA: Non-activated MΦ
LPS: Lipopolysaccharide
INF-γ: Interferon-gamma
TGF-β: Transforming growth factor-beta
CD: A cluster of differentiation
SDS-PAGE: Sodium dodecyl sulfate-polyacrylamide gel electrophoresis
∆∆Ct: Delta-delta cycle threshold
TBP: Tata-box binding protein
MCHII: Major histocompatibility complex II
TNF-α: Tumor necrosis factor α
siRNA: Small interfering RNA
iNOS2: Inducible nitric oxide 2
