# Supplementary material for: Head and neck squamous cell carcinoma cell lines have an immunomodulatory effect on macrophages independent of hypoxia and toll-like receptor 9
Source: BMC Cancer. 2021 Sep 3;21:990. doi: 10.1186/s12885-021-08357-8 (PMC8418007; doi:10.1186/s12885-021-08357-8)
Supplement: Supplementary file 1 — Additional file 1. Additional Material and Methods. TLR9-targeted CRISPR/Cas9 modification in FaDu cells. (a) mRNA analysis verified that in CRISPR/Cas9-modified FaDu cells (FaDuTLR9def) TLR9 mRNA expression under normoxia was significantly reduced with median of 0.5105 with 95% CI (± 0.1792 and 1.125) in comparison to parental FaDu cells (dotted line). Error bars represent min and max, n = 7, *p ≥ 0.05. (b) Western blot gels showing a moderate decrease of TLR9-protein expression in CRISPR/Cas9-modified FaDu cells. (c) The clone with the lowest TLR9-expression was selected for Sanger sequencing. The sequencing result confirmed that the cell line had a genetic modification in one allele only. [file 12885_2021_8357_MOESM1_ESM.pdf]

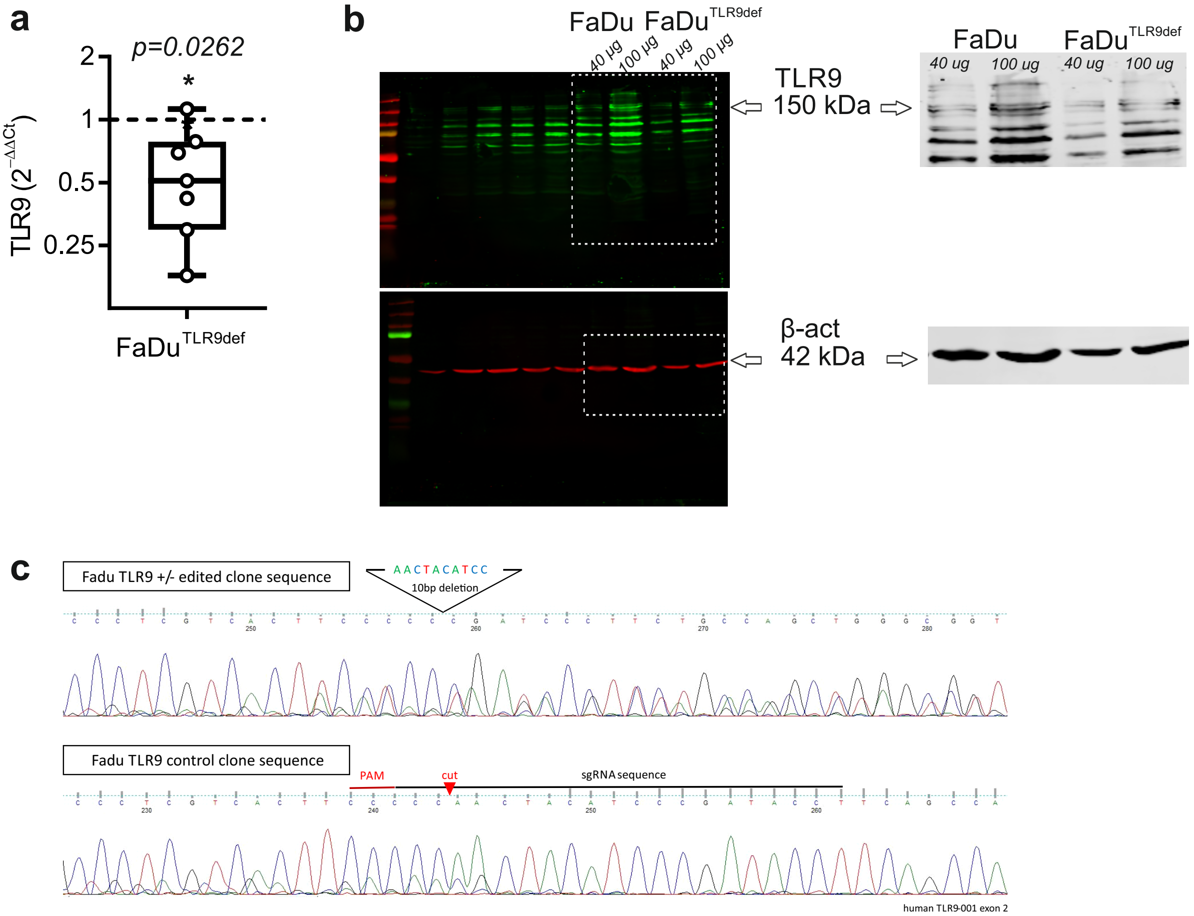

Additional Material and Methods F1. TLR9-targeted CRISPR/Cas9 modification in FaDu cells. (a) mRNA analysis verified that in CRISPR/Cas9-modified FaDu cells (FaDu^{TLR9def}) TLR9 mRNA expression under normoxia was significantly reduced with median of 0.5105 with 95% CI (± 0.1792 and 1.125) in comparison to parental FaDu cells (dotted line). Error bars represent min and max, n=7, *p≥ 0.05. (b) Western blot gels showing a moderate decrease of TLR9-protein expression in CRISPR/Cas9-modified FaDu cells. (c) The clone with the lowest TLR9-expression was selected for Sanger sequencing. The sequencing result confirmed that the cell line had a genetic modification in one allele only.
