# Supplementary material for: Head and neck squamous cell carcinoma cell lines have an immunomodulatory effect on macrophages independent of hypoxia and toll-like receptor 9
Source: BMC Cancer. 2021 Sep 3;21:990. doi: 10.1186/s12885-021-08357-8 (PMC8418007; doi:10.1186/s12885-021-08357-8)
Supplement: Supplementary file 2 — Additional file 2. The gating strategy for macrophage analysis. (a) The gating strategy of human monocytes based on their FSC/SSC profiles, following the viability marker Fixable Viability Dye eFluor 780 and anti-CD68+-based subgating. (b) Representative flow cytometry histograms show all treatment groups and controls for a particular antibody: isotype (dark green), NA (red), M1 (blue), M2a (orange), and M2c (light green).﻿ [file 12885_2021_8357_MOESM2_ESM.pdf]

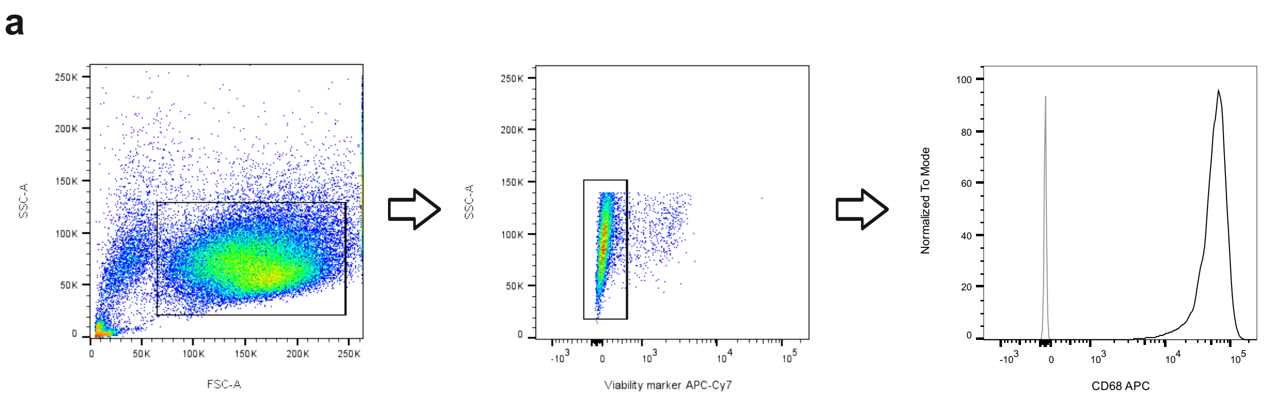

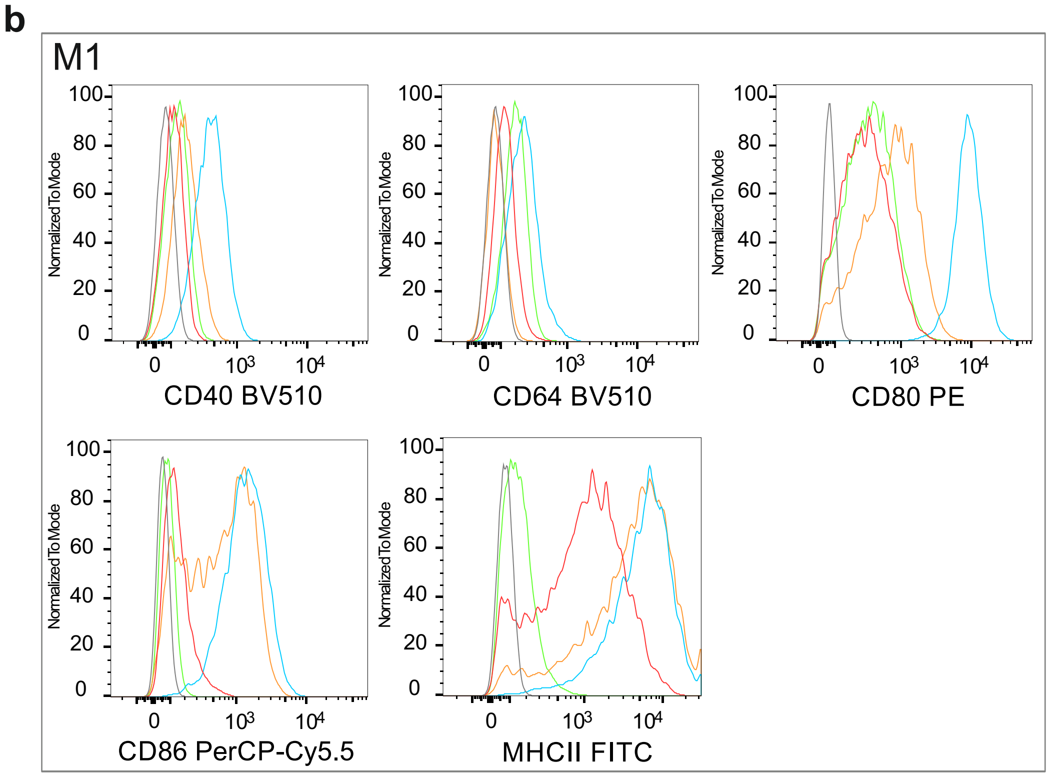

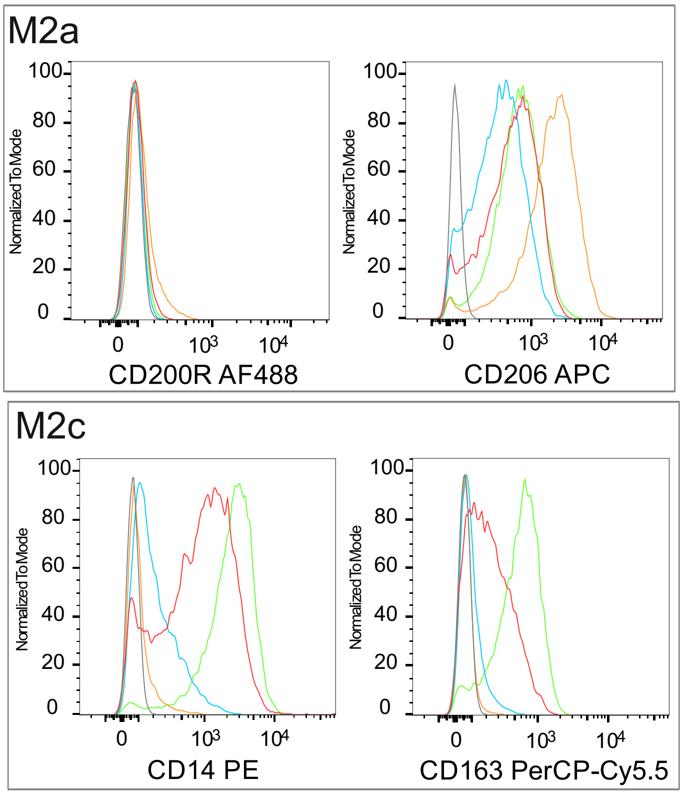

Add F2. The gating strategy for macrophage analysis. (a) The gating strategy of human monocytes based on their FSC/SSC profiles, following the viability marker Fixable Viability Dye eFluor 780 and anti-CD68^{+}-based subgating. (b) Representative flow cytometry histograms show all treatment groups and controls for a particular antibody: isotype (dark green), NA (red), M1 (blue), M2a (orange), and M2c (light green).
