# Supplementary material for: Head and neck squamous cell carcinoma cell lines have an immunomodulatory effect on macrophages independent of hypoxia and toll-like receptor 9
Source: BMC Cancer. 2021 Sep 3;21:990. doi: 10.1186/s12885-021-08357-8 (PMC8418007; doi:10.1186/s12885-021-08357-8)
Supplement: Supplementary file 3 — Additional file 3. Primers used in real-time PCR. [file 12885_2021_8357_MOESM3_ESM.pdf]

Add F3. Primers used in real-time PCR

	Gene	Primer sequences	Approximate length of PCR fragment
SYBR Green	TBP	5'-ACTTCACATCACAGCTCC-3'<br>5'-GAATATAATCCCAAGCGG-3'	191 bp
	LDH-A	5'-TCTCTGGCAAAGTGGATA-3'<br>5'-CCAGCCTTTCCCCCATTA-3'	94 bp
	TLR9	qHSACED0003672	98 bp
Taqman	IL6	Hs00985639_m1	66 bp
	IL12A	Hs01073447_m1	52 bp
	IL12B	Hs01011518_m1	72 bp
	TNFα	Hs00174128_m1	80 bp
	IL10	Hs00961622_m1	74 bp
	TGFβ	Hs00998133_m1	57 bp
	NOS2	Hs01075529_m1	67 bp
	TBP	Hs00427620_m1	91 bp
