# Supplementary material for: Head and neck squamous cell carcinoma cell lines have an immunomodulatory effect on macrophages independent of hypoxia and toll-like receptor 9
Source: BMC Cancer. 2021 Sep 3;21:990. doi: 10.1186/s12885-021-08357-8 (PMC8418007; doi:10.1186/s12885-021-08357-8)
Supplement: Supplementary file 4 — Additional file 4. Non-activated MΦ controls M1, M2a, and M2c polarization marker expressions. (a) Following incubation with control cytokines (M1: LPS + INFγ; M2a: IL4 + IL13; M2c: TGFβ+IL10) for two days, MΦ polarization was assessed with flow cytometry. Box plots represent MFI values normalized to non-activated MΦ (nMFI), error bars 95% CI. The dotted line represents non-activated MΦ. n = 5–10 independent experiments. All markers, except CD80 in M2c, were statistically significant (p ≤ 0.05). (b) Listed p-values describing the statistical significance of M1 (green), M2a (blue), and M2c (gray) marker expressions. The tint of the color signifies the direction of the expression: darker color represents a median expression (nMFI) above NA MΦ. Similarly, lighter color represents median expression below NA MΦ. [file 12885_2021_8357_MOESM4_ESM.pdf]

a

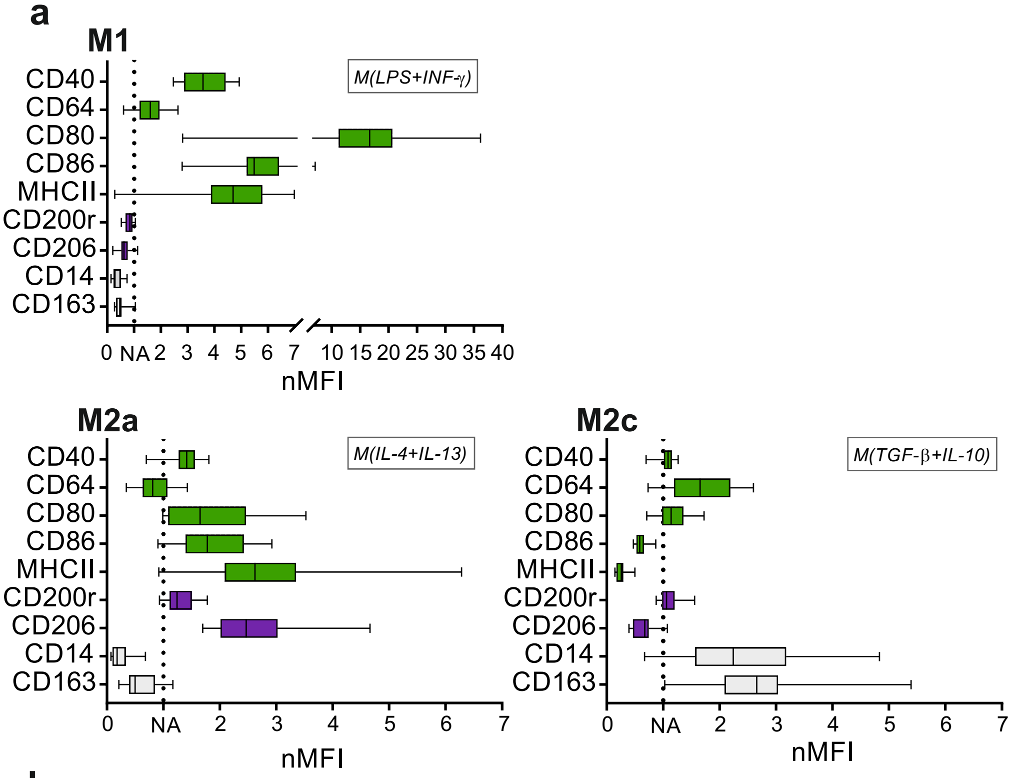

b

	nMFI	M1					M2a		M2c	
		CD40	CD64	CD80	CD86	MHCII	CD200r	CD206	CD14	CD163
M1	MEDIAN	3.577	1.611	16.66	5.485	4.696	0.848	0.623	0.29	0.469
	p	*<0.0001*	*0.0004*	*<0.0001*	*0.0002*	*<0.0001*	*0.0003*	*0.0002*	*<0.0001*	*0.0001*
M2a	MEDIAN	1.413	0.811	1.65	1.779	2.623	1.243	2.467	0.182	0.498
	p	*<0.0001*	*0.037*	*<0.0001*	*<0.0001*	*<0.0001*	*<0.0001*	*<0.0001*	*<0.0001*	*<0.0001*
M2c	MEDIAN	1.089	1.656	1.143	0.584	0.257	1.058	0.675	2.24	2.657
	p	*0.0151*	*0.0002*	*0.0577*	*<0.0001*	*<0.0001*	*0.0367*	*0.0003*	*0.0001*	*<0.0001*

Add F4. Non-activated MΦ controls M1, M2a, and M2c polarization marker expressions. (a) Following incubation with control cytokines (M1: LPS+INFγ; M2a: IL4+IL13; M2c: TGFβ+IL10) for two days, MΦ polarization was assessed with flow cytometry. Bar graphs represent MFI values normalized to non-activated MΦ (nMFI), error bars 95% CI. The dotted line represents non-activated MΦ. n = 5-10 independent experiments. All markers, except CD80 in M2c, were statistically significant ($p \leq 0.05$). (b) Listed p-values describing the statistical significance of M1 (green), M2a (blue), and M2c (gray) marker expressions. The tint of the color signifies the direction of the expression: darker color represents a median expression (nMFI) above NA MΦ. Similarly, lighter color represents median expression below NA MΦ.
