# Supplementary material for: Head and neck squamous cell carcinoma cell lines have an immunomodulatory effect on macrophages independent of hypoxia and toll-like receptor 9
Source: BMC Cancer. 2021 Sep 3;21:990. doi: 10.1186/s12885-021-08357-8 (PMC8418007; doi:10.1186/s12885-021-08357-8)
Supplement: Supplementary file 5 — Additional file 5. Cytokine mRNA median ∆Ct and statistical p-values of MΦ treated with FaDu conditioned media collected under normoxia or hypoxia (CMNOX or CMHOX). MΦ were treated with (a) conditioned media from FaDu cells collected under normoxia or hypoxia (CMNOX or CMHOX) or (b) conditioned media from FaDu or FaDuTLR9def after 48 h exposure to HOX. [file 12885_2021_8357_MOESM5_ESM.pdf]

a

IL-6	ΔCt	*p*
NA MΦ	4.739	
$FaDu_{NOX}$	2.129	*0.014*
$FaDu_{HOX}$	2.367	*0.011*

IL-12	ΔCt	*p*
NA MΦ	6.273	
$FaDu_{NOX}$	6.726	*>0.999*
$FaDu_{HOX}$	6.752	*>0.999*

TNFα	ΔCt	*p*
NA MΦ	0.029	
$FaDu_{NOX}$	0.404	*>0.999*
$FaDu_{HOX}$	0.270	*>0.999*

IL-10	ΔCt	*p*
NA MΦ	-1.491	
$FaDu_{NOX}$	-2.416	*0.012*
$FaDu_{HOX}$	-2.379	*0.020*

TGFβ	ΔCt	*p*
NA MΦ	-5.564	
$FaDu_{NOX}$	-5.897	*>0.999*
$FaDu_{HOX}$	-6.004	*>0.999*

iNOS2	ΔCt	*p*
NA MΦ	10.30	
$FaDu_{NOX}$	9.304	*0.199*
$FaDu_{HOX}$	9.124	*0.060*

b

IL-6	ΔCt	*p*	
NA MΦ	4.739		
FaDu	2.129	*0.014*	*NOX*
$FaDu^{TLR9def}$	1.867	*0.004*	*NOX*
FaDu	2.367	*0.011*	*HOX*
$FaDu^{TLR9def}$	2.070	*0.006*	*HOX*

IL-10	ΔCt	*p*	
NA MΦ	-1.491		
FaDu	-2.416	*0.011*	*NOX*
$FaDu^{TLR9def}$	-2.467	*0.003*	*NOX*
FaDu	-2.379	*0.005*	*HOX*
$FaDu^{TLR9def}$	-2.395	*0.011*	*HOX*

IL-12	ΔCt	*p*	
NA MΦ	6.273		
FaDu	6.726	*>0.9999*	*NOX*
$FaDu^{TLR9def}$	7.198	*0.163*	*NOX*
FaDu	6.752	*>0.9999*	*HOX*
$FaDu^{TLR9def}$	7.205	*0.249*	*HOX*

TGFβ	ΔCt	*p*	
NA MΦ	-5.564		
FaDu	-5.897	*0.820*	*NOX*
$FaDu^{TLR9def}$	-5.891	*0.820*	*NOX*
FaDu	-6.004	*0.596*	*HOX*
$FaDu^{TLR9def}$	-5.761	*>0.999*	*HOX*

TNFα	ΔCt	*p*	
NA MΦ	0.029		
FaDu	0.404	*>0.999*	*NOX*
$FaDu^{TLR9def}$	0.308	*>0.999*	*NOX*
FaDu	0.27	*>0.999*	*HOX*
$FaDu^{TLR9def}$	0.612	*>0.999*	*HOX*

iNOS2	ΔCt	*p*	
NA MΦ	10.30		
FaDu	9.304	*0.079*	*NOX*
$FaDu^{TLR9def}$	9.978	*0.317*	*NOX*
FaDu	9.124	*0.032*	*HOX*
$FaDu^{TLR9def}$	9.593	*0.405*	*HOX*

Add F5. Cytokine mRNA median ΔCt and statistical p-values of MΦ treated with FaDu conditioned media collected under normoxia or hypoxia (CM_{NOX} or CM_{HOX}). MΦ were treated with (a) conditioned media from FaDu cells collected under normoxia or hypoxia (CM_{NOX} or CM_{HOX}) or (b) conditioned media from FaDu or $FaDu^{TLR9def}$ after 48h exposure to HOX.
