# Supplementary material for: Head and neck squamous cell carcinoma cell lines have an immunomodulatory effect on macrophages independent of hypoxia and toll-like receptor 9
Source: BMC Cancer. 2021 Sep 3;21:990. doi: 10.1186/s12885-021-08357-8 (PMC8418007; doi:10.1186/s12885-021-08357-8)
Supplement: Supplementary file 6 — Additional file 6. Expression of TLR9 mRNA under hypoxia (a) Cancer cells were exposed to hypoxia (HOX, 1% O2), and RNA samples were collected at 24 and 48 h to assess TLR9 mRNA expression. Unlike lactate dehydrogenase-A (LDH-A) mRNA expression, TLR9 mRNA had a variable expression pattern among cell lines. The dotted line represents corresponding normoxic (21% O2) samples. Results from a minimum of 3 independent experiments are shown as box plots with 95% CI. (b) Expression of TLR9 may require input from several cooperative factors together with HIF-1α. TLR9 mRNA expression in FaDu cells increased gradually during prolonged HOX exposure. [file 12885_2021_8357_MOESM6_ESM.pdf]

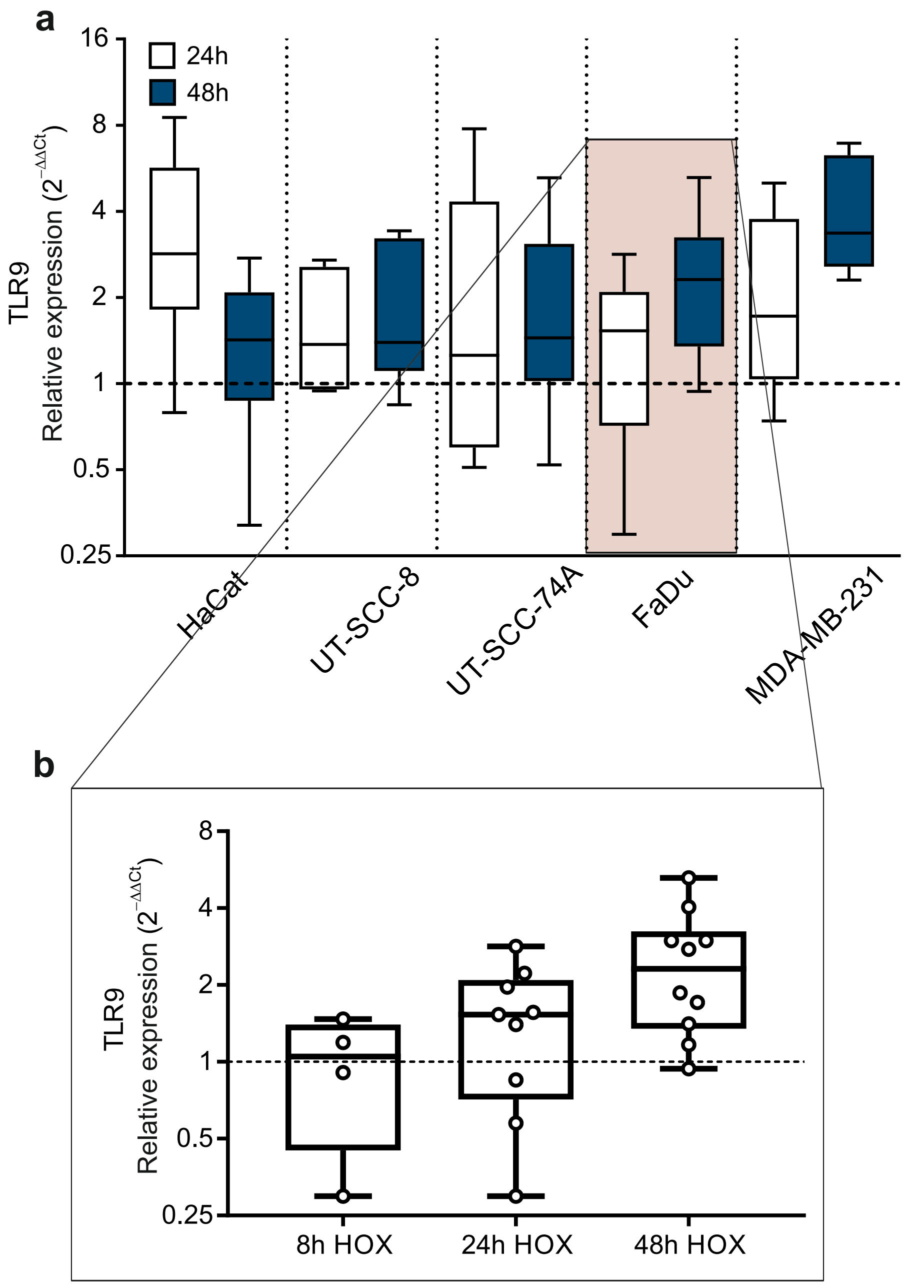

Add F6. Expression of TLR9 mRNA under hypoxia (a) Cancer cells were exposed to hypoxia (HOX, 1% O_2), and RNA samples were collected at 24 and 48 hours to assess TLR9 mRNA expression. Unlike lactate dehydrogenase-A (LDH-A) mRNA expression, TLR9 mRNA had a variable expression pattern among cell lines. The dotted line represents corresponding normoxic (21% O_2) samples. Results from a minimum of 3 independent experiments are shown as box plots with 95% CI. (b) Expression of TLR9 may require input from several cooperative factors together with HIF-1α. TLR9 mRNA expression in FaDu cells increased gradually during prolonged HOX exposure.
