# Supplementary material for: Head and neck squamous cell carcinoma cell lines have an immunomodulatory effect on macrophages independent of hypoxia and toll-like receptor 9
Source: BMC Cancer. 2021 Sep 3;21:990. doi: 10.1186/s12885-021-08357-8 (PMC8418007; doi:10.1186/s12885-021-08357-8)
Supplement: Supplementary file 7 — Additional file 7. A summary of the CM-induced MΦ polarization phenotypes (a) CM-mediated hybridized MΦ polarization phenotype, demonstrates a direct mechanism cancer undertakes to evade anti-tumorigenic immune responses. Values represent MFI values normalized to non-activated MΦ (nMFI), n = 5–10. (b) Listed p-values describing the statistical significance of M1 (green), M2a (blue), and M2c (gray) marker expressions after exposure to the variety of CMs. The tint of the color signifies the direction of the expression: darker color represents a median expression (nMFI) above NA MΦ. Similarly, lighter color represents median expression below NA MΦ. [file 12885_2021_8357_MOESM7_ESM.pdf]

a

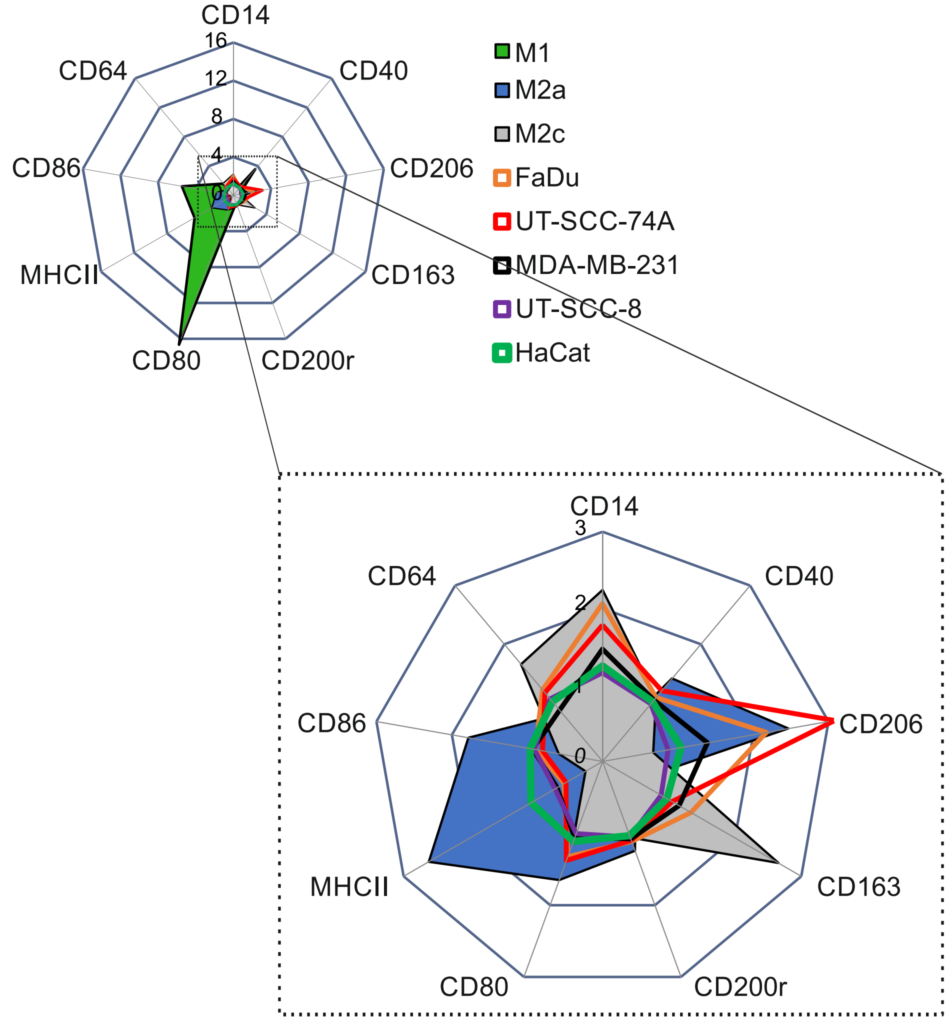

b

		M1					M2a		M2c	
		**CD40**	**CD64**	**CD80**	**CD86**	**MHCII**	**CD200r**	**CD206**	**CD14**	**CD163**
**FaDu**	**MEDIAN**(nMFI)	1.09	1.22	1.31	0.8925	0.548	1.112	1.995	2.098	1.368
	P	*0.0106*	*0.0005*	*0.0006*	*<0.0001*	*<0.0001*	*<0.0001*	*0.0068*	*<0.0001*	*<0.0001*
**UT-SCC-74A**	**MEDIAN**(nMFI)	1.209	1.213	1.396	0.791	0.5485	1.106	3.083	1.78	1.081
	P	0.0029	0.002	0.0098	0.001	0.0005	0.0371	0.0005	0.002	0.2104
**UT-SCC-8**	**MEDIAN**(nMFI)	0.982	1.068	0.898	0.9	0.7085	1.055	0.8695	1.158	1.008
	P	0.5703	0.1289	0.3125	0.0781	0.0488	0.0059	0.9219	0.0195	0.7695
**MDA-MB-231**	**MEDIAN**(nMFI)	1.047	0.862	1.062	0.926	0.686	1.079	1.393	1.467	1.158
	P	0.125	0.125	0.4375	0.3125	0.3125	0.0625	0.3125	0.0625	0.625
**HaCat**	**MEDIAN**(nMFI)	1.019	1.019	1.115	0.959	1.08	1.016	1.045	1.25	0.971
	P	0.6875	0.5	0.0156	0.1563	0.375	0.8125	>0.9999	0.2188	0.6875

Add F7. A summary of the CM-induced MΦ polarization phenotypes (a) CM-mediated hybridized MΦ polarization phenotype, demonstrates a direct mechanism cancer undertakes to evade anti-tumorigenic immune responses. Values represent MFI values normalized to non-activated MΦ (nMFI), n = 5-10. (b) Listed p-values describing the statistical significance of M1 (green), M2a (blue), and M2c (gray) marker expressions after exposure to the variety of CMs. The tint of the color signifies the direction of the expression: darker color represents a median expression (nMFI) above NA MΦ. Similarly, lighter color represents median expression below NA MΦ.
